# Microbial signaling and pest control in a medicinal plant: insights into insect, fungus, plant interactions

**DOI:** 10.3389/fmicb.2026.1767752

**Published:** 2026-10-05

**Authors:** Chao Zhang, Yinna Li, Jing He, Xiaodan Si, Linjie Zhao, Yeyun Wang, Chenyu Yang, Hongliang Wang, Songwei Li

**Affiliations:** 1 School of Plant Protection and Environment (School of Bee Science), Henan Institute of Science and Technology, Xinxiang, China; 2 Henan Province Engineering Research Center of Biological Pesticide & Fertilizer Development and Synergistic Application, Henan Institute of Science and Technology, Xinxiang, China; 3 Xinxiang County Agricultural Technology Extension Station, Xinxiang County Bureau of Agriculture and Rural Affairs, Xinxiang, China

**Keywords:** biome, entomopathogenic fungus, medicinal plant, micro, microbiology, sustainable agriculture, tri-trophic interactions

## Abstract

Tri-trophic interactions are dynamic relationships between plants, herbivores, and natural enemies and such interactions play important roles in agricultural ecosystems with regard to crop health, such as in the case of *Aloe vera* crops. *A. vera* is an agriculturally and medicinally important plant, known for its medicinal value and resilience in arid conditions. Herbivorous pests cause damage to the plant that allows entry of pathogens through infected parts and decreases the commercial and medicinal value of *A. vera*. However, there is an identified potential endophytic fungi it induces systemic resistance by eliminating pests in *A. vera* and enhances the plant defensive strategy against further pests and pathogens. Moreover, microbial signaling plays a crucial role in the Tri-trophic interactions via chemical signals enabling communication among plants, pests, and pathogens. Understanding further research into microbial signaling pathways is needed to improve biocontrol methods. The continued progress of biotechnology opens new ways to improve the efficiency of endophytic fungi and *A. vera*’s pest resistance. Tri-trophic interactions created within this framework underscore the implications of its usage in pest management under conditions of ecological stress.

## 1 Introduction

Tri-trophic interactions are relationship consisting of the multiple and intricate interconnections among three principal trophic levels in ecosystems. Ecosystems with plants as primary producers, herbivores as the first-order consumers, and natural enemies who are pathogens or predators of the herbivores as the second-order consumers are perfect examples of these intimate interactions that are inter-derived (Islam et al., 2021). The direction and effectiveness of energy flows, the distribution of species, and the rigidity of ecosystems become influenced by these interactions and are, therefore, crucial to the existence and efficiency of ecosystem structures (Yang et al., 2024). Tri-trophic levels have strong effects on crop condition, pest control, and the adaptation of eco-friendly farming methods for the agricultural production (Schausberger et al., 2021). This means the capacity of agriculture to produce a surplus of crops, to diminish pests influence, and to use less chemical pesticides is greatly affected by the proper management of the tri-trophic interactions. The potential yield can be either improved by the intervention of natural enemies (Weber et al., 2021; Clark and Crowder, 2021). For example, the presence of natural enemies like parasites or predators is the most effective method of pest control as it aids in the balance of the environment that cause diseases in plants (Ayoade et al., 2024; Sacco-Martret de Preville et al., 2024). The intensive agricultural practices employing chemical additives have increased the pesticide compounds quantity in the soil eco-profile. To date, the application of pesticides is considered adequate for protecting plant crops from various pests. But then, only1% of the pesticide reaches the pest, whereas the remaining retains in the soil, and water ecosystem, thereby entering the food chain and ultimately affecting flora, fauna, and mankind (Gaffney, 2019).

*A. vera* (which is another name of *Aloe barbadensis* Miller) is a herbaceous stem less or very short-stemmed plants and they belong in the Aloe genus of the Asphodelaceae family. It is also beneficial because it as good medicinal value and it can also thrive in a dry environment. This plant is extensively grown in areas characterized by suboptimal soil quality and minimal precipitation, flourishing as a result of its water-storing tissues and capacity to withstand environmental challenges (Skowrońska and Bazylko, 2023; Catalano et al., 2024; Kaur and Bains, 2025). Besides its therapeutic uses, *A. vera* receives the attention of agricultural scientists because of its peculiar ecological relationships and the challenges that come from herbivorous organisms and pathogens (Kaur and Bains, 2025).

Plant aphid interactions trigger complex defense responses. Aphids use specialized stylets to extract phloem sap, activating plant signaling pathways that mediate structural and biochemical defenses, including sieve tube occlusion, reactive oxygen species (ROS), and deposition of defensive compounds (Yang et al., 2024; Shikano et al., 2017). VOCs released upon herbivory can repel aphids and attract aphid predators and parasitoids, reinforcing tritrophic defense mechanisms.

Plant-microbiomes, including beneficial rhizosphere and endophytic fungi, are integral components of plant biology that influence nutrient cycling, stress tolerance, and defense against biotic threats. Endophytic microorganisms can modulate plant physiology via production of metabolites and phytohormones, contributing to enhanced tolerance to pests and diseases. Symbiotic fungi such as arbuscular mycorrhizal fungi (AMF) influence plant defense gene expression and are involved in multitrophic interactions with herbivores.

Endophytic entomopathogenic fungi can colonize host plants asymptomatically and alter host chemistry in ways that influence herbivore performance and plant defense profiles. These fungi can change plant secondary metabolites, VOC blends, and nutrient content, potentially affecting herbivore colonization and natural enemy attraction. Endophytic fungi also produce enzymes and metabolites that can directly impact herbivores or indirectly attract predators through induced plant signals.

Fungi in the genus *Aspergillus* are widely distributed in plant ecosystems, and many species harbor biosynthetic gene clusters that produce diverse bioactive compounds, including alkaloids and phenolics with potential antimicrobial and insecticidal propertie. Laboratory studies have demonstrated insecticidal effects of metabolites from *A. niger* associated extracts against insect pests (Wu et al., 2012), indicating that *Aspergillus* metabolites may contribute to pest suppression in some systems (e.g., aspernigerin against *Plutella xylostella*).

Plant defense signaling pathways, notably jasmonic acid (JA) and salicylic acid (SA)—regulate responses to both herbivore feeding and microbial elicitors, integrating environmental signals to tailor defense outputs. Phytohormone modulation by microbial endophytes has been documented in multiple plant species, showing impacts on growth and inducible defense responses.

Previous studies across plant, microbe, and herbivore research demonstrates that Tri-trophic interactions in crops are mediated by plant signaling and microbial influences. Endophytic fungi alter host physiology and secondary chemistry, affect insect herbivore performance, and modulate ecological signaling that contributes to natural enemy recruitment. These multi, level interactions underscore the ecological complexity of plant–herbivore–microbe systems and inform sustainable pest management approaches.

## 2 *Aloe vera* ecosystem, pests, and biocontrol

*Aloe vera* (syn. *Aloe barbadensis* Mill.) is a perennial succulent plant belonging to the family Xanthorrhoeaceae and is widely cultivated for its medicinal, cosmetic, and nutraceutical applications. The plant is native to arid and semi-arid regions of North Africa, the Middle East, the southern Mediterranean, and the Canary Islands. Its thick, fleshy leaves store water and contain a mucilaginous gel rich in polysaccharides, anthraquinones, vitamins, and phenolic compounds that are extensively documented for wound healing, anti-inflammatory, antioxidant, and antimicrobial properties (Eshun and He, 2004; Hamman, 2008; Surjushe et al., 2008).

Despite its tolerance to drought and poor soils, *A. vera* supports diverse biological communities and is integrated into complex agro-ecosystems (Table 1). The rhizosphere and phyllosphere of *A. vera* harbor structurally diverse microbial communities, including bacteria, fungi, and archaea, that contribute directly to nutrient cycling and plant health. High, throughput sequencing and culture, dependent studies have consistently identified Proteobacteria, Firmicutes, Actinobacteria, and Bacteroidetes as dominant bacterial phyla associated with *A. vera* tissues (Akinsanya et al., 2015). These microbial assemblages are shaped by plant tissue type and soil conditions and are functionally involved in nitrogen cycling, phosphate solubilization, and organic matter turnover (Koch and Sessitsch, 2024).

**Table 1:** Overview of the *Aloe vera* agro-ecosystem and tri-trophic interactions.

Component	Trophic/ecological role	Examples	Impact on *Aloe vera* and tri-trophic interactions	Reference
*Aloe vera* plant	Primary producer	Succulent medicinal crop; source of polysaccharides, phenolics, anthraquinones	Provides resources for herbivores and microbes; activates inducible defenses (JA, SA signaling), produces secondary metabolites and VOCs that mediate herbivore deterrence and natural enemy attraction	Skowrońska andBazylko (2023), Kaur and Bains (2025), and Yang et al. (2024)
Herbivorous pests	Primary consumers	Aphids (*Aphis gossypii*)	Phloem sap feeding reduces photosynthesis and plant vigor; triggers plant defense signaling (ROS, sieve tube occlusion, secondary metabolites); facilitates pathogen entry	Blackman and Eastop, (2020)
Soil and plant microbiome	Mediators of nutrient cycling and plant defense	Rhizosphere bacteria (Proteobacteria, Actinobacteria), endophytes (Bacillus, Pseudomonas), AMF (Funneliformis, Rhizophagus)	Enhances nutrient acquisition, stress tolerance, and growth; modulates phytohormone signaling; primes plant defenses and indirectly affects herbivore performance and natural enemy recruitment	Akinsanya et al. (2015), and Koch and Sessitsch (2024)
Endophytic and entomopathogenic fungi	Microbial regulators of herbivore populations	*Aspergillus niger*, AMF, entomopathogenic endophytes	Produce bioactive metabolites with insecticidal or deterrent properties; alter plant secondary chemistry and VOC profiles; reduce herbivore fitness and enhance top-down control	Rapparini et al. (2024)
Signaling molecules	Chemical mediators of tri-trophic interactions	Jasmonic acid (JA), salicylic acid (SA), VOCs, microbial elicitors	Integrate herbivore and microbial cues; regulate plant defense gene expression; attract predators and parasitoids; influence herbivore behavior	Pieterse et al. (2012), and Noman et al. (2020)
Natural enemies	Secondary consumers (biocontrol agents)	Lady beetles, predatory beetles, parasitoids	Suppress aphid populations; reduce reliance on chemical pesticides; stabilize ecosystem dynamics through top-down regulation	Weber et al. (2021), Clark and Crowder (2021), and Ayoade et al. (2024)
Environmental factors	Modulators of ecosystem processes	Soil type, temperature, humidity, irrigation	Influence microbial diversity, plant physiology, pest pressure, and efficacy of natural enemies and microbial biocontrol agents	Nadarajah and Abdul Rahman (2023)

Symbiotic associations with arbuscular mycorrhizal fungi (AMF) are well documented in *A. vera* cultivation systems. Experimental inoculation with AMF species such as *Funneliformis mosseae* and *Rhizophagus intraradices* has been shown to enhance nutrient uptake, root biomass, and overall plant growth under both normal and water limited conditions. These associations improve phosphorus and nitrogen acquisition and increase photosynthetic efficiency, thereby contributing to plant productivity in nutrient poor soils.

Endophytic bacteria isolated from *A. vera* tissues, including species of *Bacillus*, *Pseudomonas*, *Enterobacter*, and *Micrococcus*, have been experimentally shown to produce antimicrobial metabolites and enzymes that inhibit plant pathogenic fungi and bacteria *in vitro* (Akinsanya et al., 2015). These findings establish the presence of functionally active microbial endophytes within *A. vera* tissues and their role in plant–microbe interactions (Figure 1).

**Figure 1:**
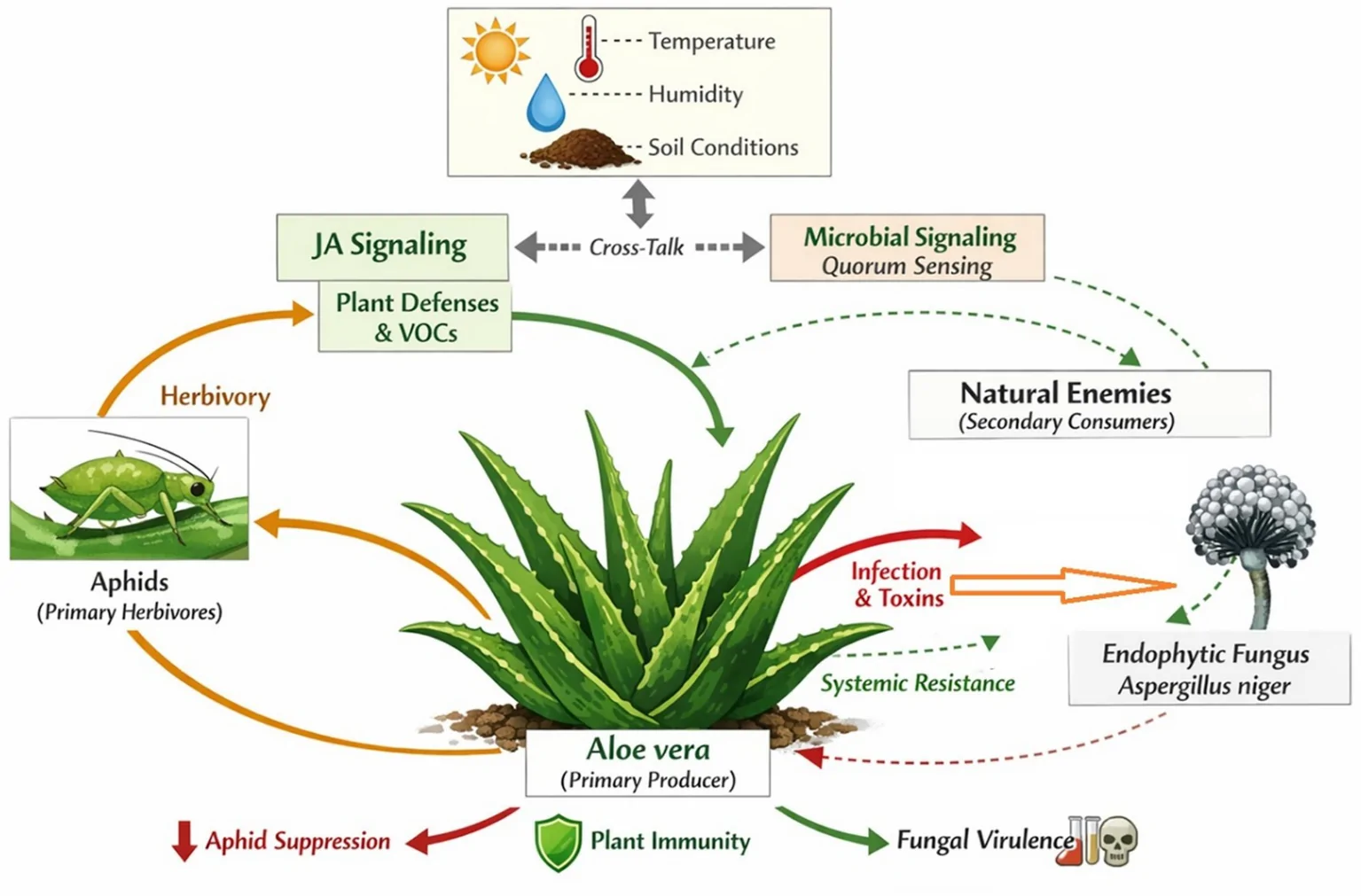
Tri-trophic interactions in the *Aloe vera* agroecosystem.

Among insect pests, aphids are recognized as economically important herbivores affecting *A. vera* cultivation. *A. gossypii* is a polyphagous sap feeding aphid species documented on succulent and medicinal crops. Aphid feeding damages *A. vera* by removing phloem sap, reducing photosynthetic capacity, and inducing localized tissue deformation. Feeding wounds caused by aphid stylets facilitate the entry of opportunistic pathogens, which further compromise leaf quality and commercial value (Blackman and Eastop, 2020).

Plant responses to aphid feeding involve well characterized defense mechanisms, including changes in secondary metabolite profiles and activation of defense, related genes. Aphid induced responses have been experimentally linked to salicylic acid dependent signaling pathways in multiple plant species, including medicinal crops, and these responses are associated with altered plant physiology and resistance traits (Pieterse et al., 2012). These defense responses represent established plant strategies for mitigating herbivore damage (Figure 2).

**Figure 2:**
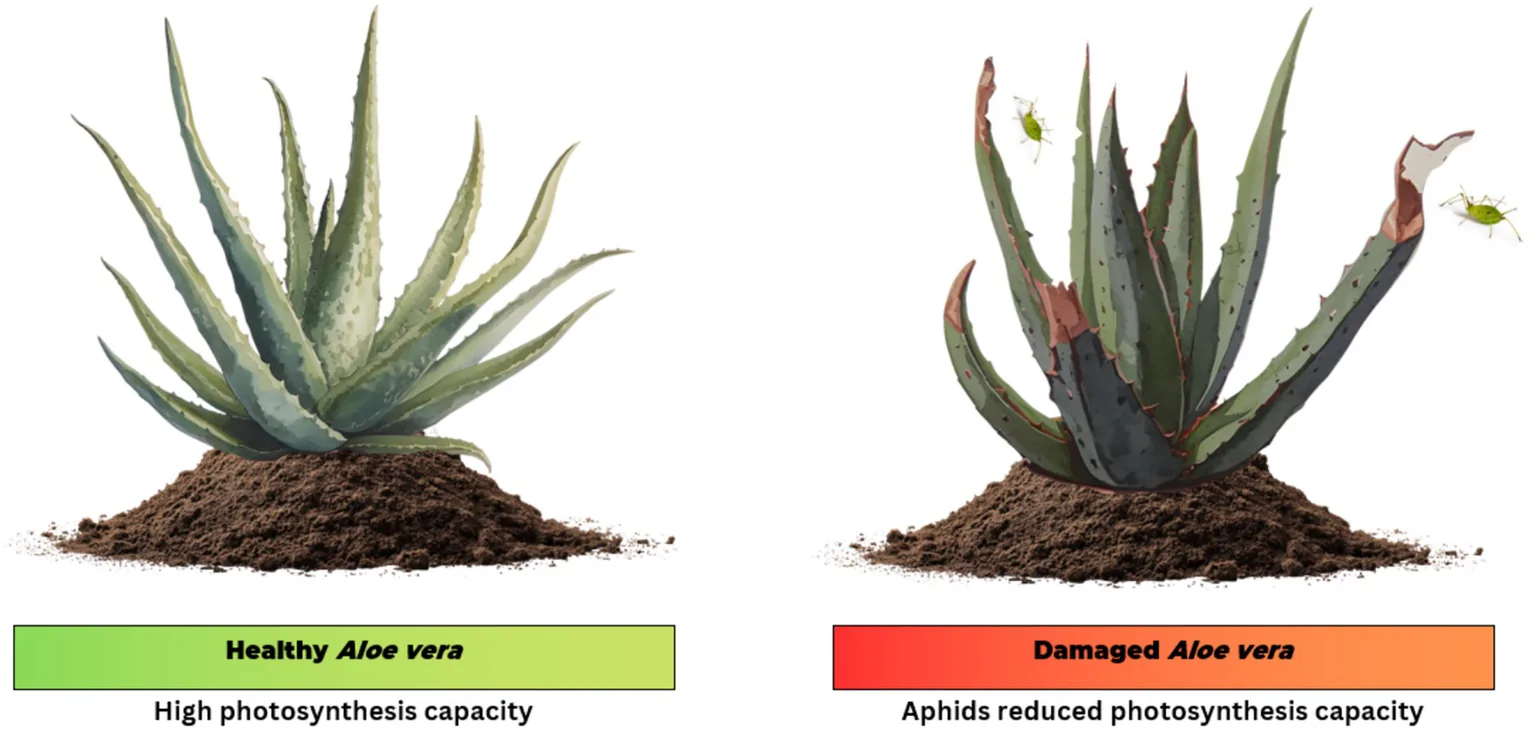
Impact of aphid feeding on *Aloe vera* plants.

Soil microbial communities surrounding *A. vera* play a direct role in sustaining plant productivity by decomposing organic matter and releasing essential nutrients such as nitrogen, phosphorus, and potassium. Soil bacteria and fungi mediate these processes through enzymatic mineralization pathways, which have been quantified in agricultural soils supporting medicinal plants (Koch and Sessitsch, 2024). Variations in soil type, irrigation practices, and fertilization regimes have been shown to significantly influence microbial diversity and plant health outcomes in *A. vera* cultivation systems (Nadarajah and Abdul Rahman, 2023).

Beyond agricultural value, *A. vera* contributes to ecosystem structure by supporting pollinators and arthropod communities. Its flowers provide nectar resources for bees and other pollinating insects, and field observations confirm its role in sustaining biodiversity in semi, arid landscapes (Akinsanya et al., 2015; Fouché et al., 2020). The plant is also used in land restoration programs, where its physiological traits support soil stabilization and vegetation recovery under degraded environmental conditions. Together, these findings demonstrate that *A. vera* ecosystems are shaped by documented interactions among plant physiology, microbial communities, and herbivorous pests (Table 2). These interactions form an ecologically grounded basis for integrated pest and soil management strategies in medicinal plant agriculture.

**Table 2:** Host range and effects of *Aspergillus niger.*

Host	Common hosts/targets	Type of interaction/effect	Reference
Heteroptera	Red cotton bug (Dysdercus koenigii)	Topical *Aspergillus niger* spores caused ~66.66% adult mortality at 1 × 10^6^ spores/mL; impacts on immune parameters (hemocytes, enzymes) documented	Kumari et al. (2019)
Lepidoptera	*Spodoptera litura* (armyworm)	Ethyl acetate extracts of *Aspergillus niger* metabolites adversely affected larval survival, development, and growth; delayed development and higher mortality observed	Kaur et al. (2016)
Diptera	Mosquito larvae (e.g., Anopheles, Aedes, Culex)	Culture filtrates of *Aspergillus niger* reduced survival of mosquito larvae (LC50/LC90 reported), indicating larvicidal activity under controlled conditions	Singh (2012)
Lepidoptera	Galleria mellonella larvae	*Aspergillus niger* α-1-3-glucan modulates immune responses by inhibiting phenoloxidase activity; demonstrates measurable effects on insect innate immunity	Stączek et al. (2020)
Nematoda (non-insect)	Meloidogyne incognita (root-knot nematode)	*Aspergillus niger* culture extracts showed high mortality and egg hatching inhibition in plant-parasitic nematodes (not an insect order but arthropod-related impact)	Niu and Liu (2023)
Diptera (vector mosquito)	Anopheles stephensi, *Culex quinquefasciatus*, *Aedes aegypti*	Filtrate can reduce larval survival in vector mosquitoes under experimental exposure conditions	Singh (2012)
Arthropod immune system	Galleria mellonella systemic immune factors	*Aspergillus niger* cell wall *α*-1-3-glucan involved in manipulation or interaction with insect innate immune pathways (phenoloxidase)	Stączek et al. (2020)

## 3 Aphids in *Aloe vera* ecosystems as herbivore

Aphids are among the most important herbivorous insects affecting medicinal and horticultural crops and are recognized as damaging pests in succulent plant systems, including *A. vera* (*A. vera*). Species such as *A. gossypii* are highly polyphagous, widely distributed, and capable of rapid population growth under favorable conditions (Blackman and Eastop, 2020; Sarwar, 2021). Aphids feed by inserting their stylets into phloem tissues and extracting sap, leading to direct depletion of photoassimilates and disruption of vascular function (Douglas, 2006; Dedryver et al., 2010). Sustained aphid feeding results in reduced plant vigor, decreased chlorophyll content, and impaired photosynthetic efficiency, effects that are well documented across multiple crop systems, including medicinal plants (Züst and Agrawal, 2016; Nalam et al., 2019).

In *A. vera*, aphid feeding damages leaf tissues, that are commercially valuable due to their gel content. Aphid infestation also leads to honeydew deposition on leaf surfaces, which supports the growth of sooty mold fungi and further reduces photosynthetic capacity by limiting light penetration and gas exchange. The damaged plant shows infected leaves, stunted growth, and signs of infection (Figure 3), with reduced photosynthetic capacity (Sharma and Bhattacherjee, 2013). These effects directly compromise leaf quality and reduce the medicinal and commercial value of *A. vera* crops (Sarwar, 2021).

**Figure 3:**
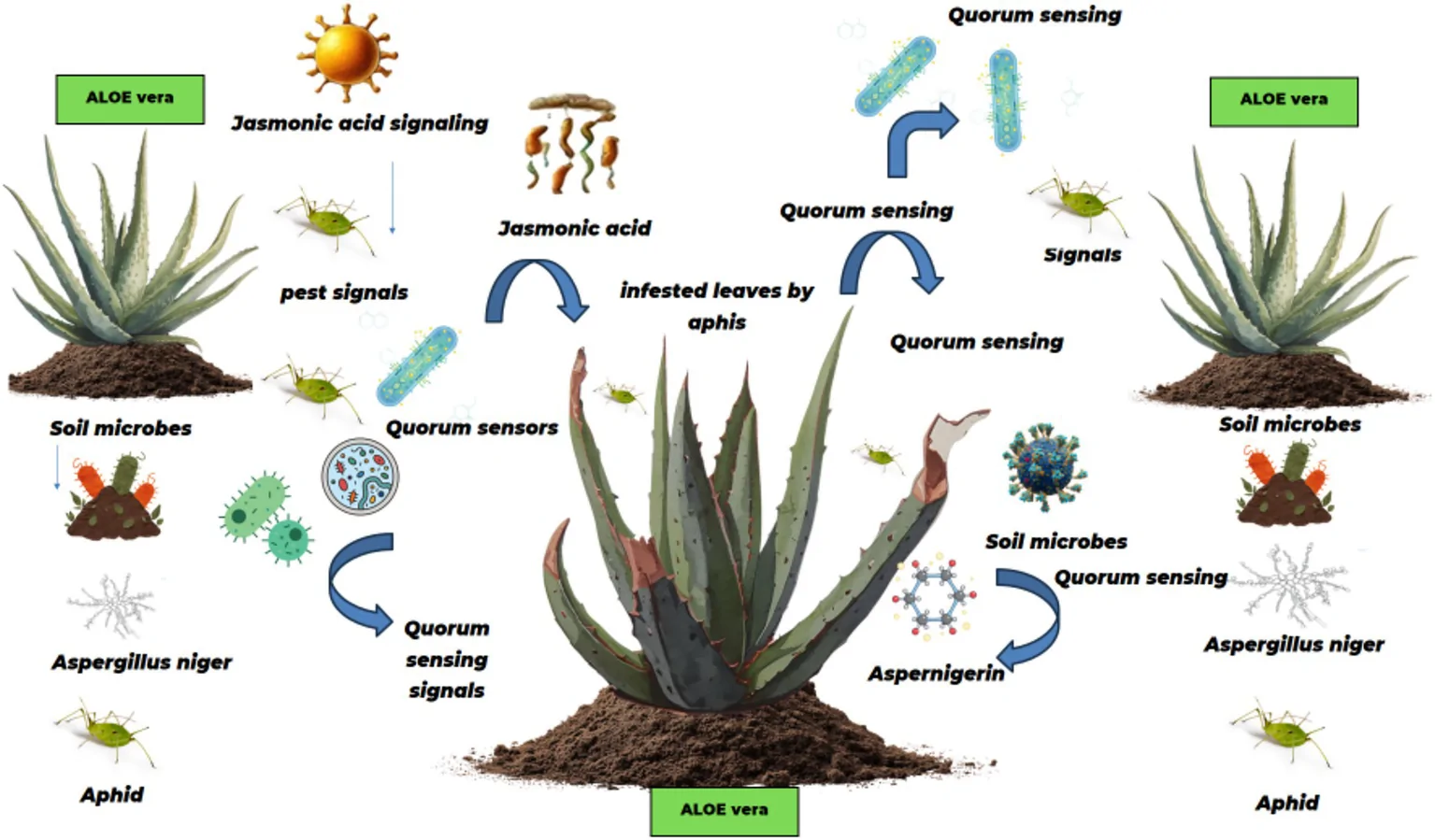
Microbial signaling pathways in *Aloe vera* tri-trophic interactions.

Endophytic fungi are consistently reported as components of plant, associated microbial communities and are defined as fungi that colonize internal plant tissues without causing visible disease symptoms. Members of the genus *Aspergillus*, including *A. niger*, have been isolated as endophytes from a wide range of plant species, including medicinal plants and succulents (Schulz and Boyle, 2005). *A. niger* is a well, characterized filamentous fungus with a documented capacity to colonize plant tissues and produce diverse secondary metabolites and extracellular enzymes.

Several studies have reported the isolation of *A. niger* as an endophytic fungus from medicinal plants, including species used in traditional medicine, confirming its ability to persist within plant tissues without inducing pathogenic symptoms. These studies establish *A. niger* as a legitimate component of plant endophytic communities, including those associated with medicinal crops.

The insecticidal activity of *A. niger* has been demonstrated in controlled laboratory studies, where metabolites and enzyme complexes produced by the fungus caused mortality or reduced performance in aphid species, including *A. gossypii* and related taxa (Vega, 2018). Reported mechanisms include cuticle degradation by hydrolytic enzymes and the production of organic acids and secondary metabolites with toxic effects on insects. These findings demonstrate that *A. niger* possesses bioactive properties relevant to aphid suppression.

In plant-insect systems, endophytic fungi can influence herbivore performance through changes in host plant chemistry resulting from fungal colonization (Hartley and Gange, 2009). Experimental evidence from non-*Aloe* plant systems shows that endophytic fungi alter concentrations of phenolics, organic acids, and other metabolites that affect aphid feeding and reproduction (Hartley et al., 2015). While *A. niger*–mediated effects on aphids have not yet been experimentally confirmed in *A. vera*, its documented endophytic lifestyle and insecticidal metabolite production provide a biologically grounded framework for investigating such interactions.

Plant defense responses to aphid feeding involve well, characterized signaling pathways, particularly salicylic acid–dependent defenses, which regulate gene expression and secondary metabolite synthesis in response to sap-feeding insects (Pieterse et al., 2012). Although hormonal signaling induced by *A. niger* has not been demonstrated in *A. vera*, studies in other plant–endophyte systems show that endophytic fungi influence host defense, related metabolic pathways.

## 4 *Aspergillus niger* in pest control and its interaction with *Aloe vera* aphids

Within the Tri-trophic framework outlined in the Introduction, the *A. vera*–aphid–*A. niger* interaction represents a conceptually defined system grounded in experimentally verified components: aphids as herbivores of medicinal plants, *A. niger* as a plant endophyte, and fungal metabolites with documented aphid toxicity. Clarifying whether endophytic *A. niger* directly influences aphid performance in *A. vera* requires targeted experimental validation, including controlled inoculation and aphid bioassays.

Endophytic fungi, such as *A. niger* (*A. niger*), are a specialized group of fungi that naturally colonize plant tissues without causing visible harm to the host plant. *A. niger*, in particular, has emerged as an important biocontrol agent due to its ability to infect and suppress insect pests, offering a sustainable alternative to chemical pesticides. As an endophyte, *A. niger* provides long, term protection against herbivorous pests by establishing a symbiotic relationship with its host plant, and recent studies have highlighted its potential in integrated pest management systems.

While traditionally viewed as a plant pathogen, *A. niger* has a broad range of insecticidal properties, particularly against sap-feeding insects such as aphids. In fact, approximately 1,000 species of entomopathogenic fungi have been identified, and *A. niger*, with its ability to colonize plant tissues, represents a novel and eco, friendly method for pest control, especially in crops like *A. vera*. Endophytic fungi are advantageous because they do not require repeated applications and can be present in plants over extended periods, ensuring continuous protection from pests such as the *A. vera* aphid (*A. gossypii*).

### 4.1 Life cycle of *Aspergillus niger*

The lifecycle of *A. niger* is critical to its potential as a biocontrol agent. The process begins with the fungal spores or conidia colonizing the interior of the host plant tissues, such as the roots, stems, or leaves. Upon infection, the fungus grows intracellular or in the intercellular spaces, producing secondary metabolites that can directly influence herbivorous insects. These metabolites include mycotoxins, which can suppress insect feeding and inhibit their reproduction. In the case of *A. vera* aphids, *A. niger* has been shown to induce toxicity and reduce aphid populations by producing toxic compounds such as ochratoxin A. Once the aphid feeds on the plant, it is exposed to the fungal toxins, which impair its feeding behavior and development, ultimately leading to its death. This highlights a crucial aspect of the relationship between *A. niger* and the plant–insect interaction: the fungus not only protects the plant but also disrupts the herbivore’s life cycle. Once the aphid population is suppressed, the fungal spores are released back into the environment, continuing the cycle of infection and pest control.

### 4.2 *Aspergillus niger* and its impact on *Aloe vera* aphid populations

*Aspergillus niger* has demonstrated effectiveness against several insect species, including those that infest *Aloe vera*. The fungus infects the aphids through several mechanisms. Initially, *A. niger* spores adhere to the surface of aphid cuticles, facilitated by the hydrophobic interactions between fungal conidia and the insect’s waxy exoskeleton. Upon attachment, the spores germinate and secrete enzymes such as lipases, chitinases, and proteases to penetrate the insect’s cuticle. The fungus then proliferates inside the aphid’s body, releasing metabolites that disrupt normal physiological functions and result in death. A key feature of the interaction between *A. niger* and aphids is the ability of the fungus to alter the aphid’s feeding behavior. *A. niger* produces several secondary metabolites (Table 3), including antifungal and insecticidal compounds, which help to reduce the aphid’s ability to damage the plant. For instance, the production of ochratoxin A and other bioactive metabolites significantly reduces aphid feeding, providing a passive form of defense to the *A. vera* plant.

**Table 3:** Secondary metabolites from *Aspergillus niger* and their insecticidal or behavioral activities.

Metabolite	Chemical class	Type of activity relevant to insects	Source	References
Aspernigerin	Alkaloid/Bioactive secondary metabolite	Bioactive against *Plutella xylostell*a (insecticidal)	Produced by *Aspergillus niger* from plant endophytes; mortality ~75% at 200 μg/mL	Wu et al. (2012)
Organic acids (citric acid, oxalic acid)	Organic acid	Can create unfavorable gut conditions for small invertebrates; nematocidal activity	Produced by *Aspergillus niger*; oxalic acid complex active against nematodes	Niu and Liu (2023)
Fatty acids and derivatives (hexadecanoic acid, octadecadienoic acid)	Fatty acids	Membrane disruption; insecticidal/repellent properties	Identified in *Aspergillus niger* VOC profiles and extract analyses	Niu and Liu (2023)
Terpenic compounds/hydrocarbons (2-methyl-6-phenyl-1,6-heptadiene)	Terpenoid/Hydrocarbon	⬜ Influence insect olfactory behavior	Produced by *Aspergillus niger*; VOCs affect insect behavior	Bianco et al. (2014).
Aflavinines	Alkaloid/Sclerotial metabolite	⬜ Anti-insectan	Found in some *Aspergillus niger* sclerotia; anti-insectan class members reported	Frisvad et al. (2018).
Polyketides/Nigerasperone A/Aurasperones	Polyketide	⬜ Broad enzyme inhibitory effects; may affect insect physiology	Bioactive compounds from *Aspergillus niger* extracts	Niu and Liu (2023)
Phenolic compounds	Phenolic	⬜ Interact with biological membranes; inhibit feeding or development	Fungal phenolic extracts from *Aspergillus niger*	Frisvad et al. (2018).
Volatile alcohols (1-octen-3-ol, 3-methyl-1-butanol)	Alcohol/VOC	⬜ Influence insect behavior (attraction/repellence)	Documented in *Aspergillus niger* volatilome	Bianco et al. (2014).

The efficacy of *A. niger* as a biocontrol agent against aphids is influenced by several environmental factors (Table 4). Similar to other fungi, temperature, humidity, and light exposure play crucial roles in its effectiveness. *A. niger* generally thrives in moderate temperatures (20–30 °C) and high humidity, conditions favorable to both the growth of the fungus and the activity of the aphids. However, UV radiation can degrade fungal spores, reducing their viability. To overcome this challenge, several strategies, such as applying UV, resistant strains or introducing the fungus during the evening or under shaded conditions, have been explored to improve the success of biocontrol applications.

**Table 4:** Impact of herbivory and environmental factors on *Aloe vera* and pest control.

Factor/condition	Effect on *Aloe vera*	Effect on entophytic fungi	Effect on pest control	Reference
Temperature (< 20 °C)	Decreased JA production, increased vulnerability to pests	Reduced fungal sporulation, less virulent	Less effective pest control due to lower fungal activity	Azarfam et al. (2020)
Temperature (20, 30 °C)	Optimal JA production, enhanced VOCs, improved indirect defenses	Optimal growth and virulence, effective pest control	Maximum biocontrol effectiveness, enhanced plant defenses	Jangra et al. (2022) and Mann and Davis, (2020)
Temperature (> 30 °C)	Reduced plant vigor, potential increase in VOCs	Inhibited fungal growth, lower virulence	Fungal activity declines, less effective pest control	Kumari and Kumar Roy (2019)
Humidity (< 60%)	Increased transpiration, reduced plant defense responses	Reduced spore viability, lower infection rates	Less fungal infection, reduced biocontrol efficacy	Hazrati et al. (2020)
Humidity (60, 80%)	Balanced water retention, optimal defense response	Optimal spore germination and fungal virulence	High fungal infection rates, effective pest suppression	López et al. (2022)
Humidity (> 80%)	Reduced transpiration, risk of microbial pathogens	Risk of fungal competition, potential spore degradation	Excess moisture can lead to microbial imbalances and less effective pest control	Hazrati et al. (2020)
Soil Quality (High organic content)	Enhanced nutrient uptake, increased defense compound synthesis	Enhanced fungal persistence, better infection rates	Higher biocontrol success due to improved soil conditions	Chowdhury et al., (2020)
Soil Quality (Low organic content)	Nutrient deficiencies, poor plant health	Reduced fungal persistence and efficacy	Reduced biocontrol potential, less effective pest suppression	Boukour et al. (2024)
Soil pH (Neutral to slightly acidic)	Balanced nutrient availability, improved plant health	Optimal fungal growth and persistence	Best biocontrol outcomes, better plant, pest dynamics	Luligo et al. (2024)
Soil quality (Salinity or drought)	Decreased water retention, stressed plants	Reduced fungal effectiveness under stress conditions	Diminished biocontrol success due to plant and fungal stress	Takabayashi (2022)

In practical terms, the use of *A. niger* as a biocontrol agent may be optimized by adjusting environmental variables to ensure fungal survival and by monitoring aphid populations to determine the most effective timing for fungal application. Moreover, researchers are investigating the potential synergistic effects of combining fungal inoculation with other biocontrol agents, such as predatory insects or plant, based repellents, to further enhance pest suppression. Therefore, the endophytic fungus *A. Niger* shows great promise as a biocontrol agent for managing aphid populations on *A. vera*. Its ability to colonize plant tissues and produce bioactive compounds that affect insect pests makes it a highly effective and environmentally friendly alternative to chemical pesticides. Continued research into the molecular mechanisms of *A. niger*’s interaction with aphids, as well as the development of more resilient strains, will likely enhance its use in sustainable pest management systems.

## 5 Microbial signaling and tri-trophic interactions

Microbial signaling refers to the biochemical pathways through which plants and associated microorganisms perceive and respond to environmental and biological stimuli. In plant–herbivore systems, phytohormones, especially salicylic acid (SA) and jasmonic acid (JA), mediate defense signaling in response to herbivory and microbial interactions. These hormones regulate plant defense gene expression and metabolic profiles when plants are challenged by herbivores or microbes. Studies in multiple plant species demonstrate that phloem-feeding insects such as aphids activate the SA signaling pathway, which often acts antagonistically to JA pathways involved in defenses against other types of attackers (Johnson et al., 2020).

*A. vera* is a medicinal succulent known to host a variety of endophytic fungi—fungi that reside in plant tissues without causing visible disease symptoms. Recent isolation studies have documented *A. niger* among the endophytic fungi present in *A. vera* leaves, indicating that *A. niger* is a consistent member of the *A. vera* microbial community. In one study, *A. niger* accounted for approximately 13.33% of endophytic fungi recovered from *A. vera* leaves by morphological and molecular methods.

*A. niger* is a filamentous mold with extensive metabolic diversity, capable of synthesizing a broad range of secondary metabolites. Genomic analyses and reviews of *A. niger* metabolism highlight its large repertoire of biosynthetic gene clusters responsible for producing diverse small molecules, including organic acids, aromatic compounds, and polyketide derivatives These metabolites are well documented for bioactivity in agricultural, industrial, and microbiological applications, including antimicrobial activity, enzyme modulation, and complex organic acid production. While much of *A. niger* research focuses on industrial biotechnology and enzymology, this metabolic versatility underpins its production of diverse chemical compounds that can influence plant or microbial systems.

As a genus, Aspergillus produces secondary metabolites that include insect bioactives. For example, the compound aspernigerin produced by *A. niger* isolates has been experimentally shown to exhibit insecticidal activity against the lepidopteran pest Plutella xylostella, causing significant mortality in controlled assays. This provides documented evidence that metabolic products from *A. niger* can affect herbivorous insect physiology under laboratory conditions, although the mode of action and broader ecological roles remain defined for specific compounds, not entire ecological interactions.

Aphids such as *A. gossypii* are widely studied phloem-feeding herbivores. Research across plant species shows that aphid feeding triggers plant defense responses involving changes to phytohormone levels and activation of defense genes. The SA pathway is typically associated with defense against phloem feeders and biotrophic pathogens, while JA pathways play roles in defense against tissue, damaging herbivores. The antagonistic dynamics between SA and JA pathways are well documented in studies of plant signaling under aphid attack.

Plant defenses also encompass the production of volatile organic compounds (VOCs) and other secondary metabolites when herbivore damage occurs. Herbivore, induced VOC emissions have been shown to mediate interactions with natural enemies and influence herbivore behavior in plant systems, reflecting a well, established signaling function of plant metabolites under attack. Although specific VOC profiles in *A. vera* under aphid herbivory are currently limited in the literature, analogous signaling mechanisms involving phytohormones and secondary metabolite regulation in other plants establish a conservative model for interpreting *A. vera* responses.

Endophytic fungi produce extracellular enzymes and bioactive compounds that contribute to their ecological roles within plant tissues. Investigations of *Aspergillus nigera. Niger* isolates from plant hosts document the production of hydrolytic enzymes such as lipases, proteases, cellulases, and others, indicating that fungal metabolism actively interacts with host biochemistry and can modify biochemical environments inside plant tissues.

This established evidence from plant defense signaling, *A. vera* endophytic colonization by *A. niger*, aphid herbivory responses, and fungal secondary metabolism collectively describes the biochemical components of Tri-trophic interactions in plant–herbivore–microbe systems without speculative assertions. These documented molecular and metabolic processes underlie the chemical communication pathways that shape ecological outcomes in such interactions.

Microbial signaling refers to the biochemical pathways by which plants and their associated microbes perceive and respond to herbivore attack, leading to the activation of defense responses. In plants, phytohormones such as jasmonic acid (JA) and salicylic acid (SA) are central regulators of defenses against herbivorous insects and microbial interactions (Figure 3). JA- mediated signaling is broadly implicated in regulating plant responses to insect feeding and in inducing defensive metabolites, while SA predominantly contributes to responses against phloem feeding insects such as aphids (Aphis spp.) and biotrophic pathogens (phytohormone signaling reviews).

In the context of herbivory, aphid feeding rapidly triggers plant defense signaling cascades that include accumulation of SA and activation of JA related pathways in localized tissues surrounding feeding sites, confirming a role for these hormones in defensive signal transduction against phloem feeders. These signaling events regulate the expression of defense related genes and lead to biosynthesis of secondary metabolites that deter further herbivory and reduce feeding success (molecular mechanisms reviews).

*A. vera* harbors diverse endophytic fungi within its tissues. Endophytes asymptomatically colonize plant organs and can produce bioactive compounds that influence plant biochemical profiles. Species within the genus Aspergillus, including *Aspergillus nigera. Niger*, are commonly detected as endophytic fungi in plant hosts and possess a large repertoire of biosynthetic gene clusters encoding enzymes for the production of diverse secondary metabolites such as organic acids, terpenoids, alkaloids, and polyketides (review of *A. niger* metabolites). While *A. niger* has been identified in association with plant tissues in non, *Aloe* systems, such fungal endophytic associations illustrate the capacity of this fungus to contribute metabolically to host plants.

Secondary metabolites from Aspergillus endophytes include compounds with documented bioactivities against insect pests. For example, *A. niger* associated with *Cynodon dactylon* produces the metabolite aspernigerin, which displayed insecticidal activity against *Plutella xylostella* larvae in controlled assays, indicating that *A. niger* and related endophytes produce metabolites with insecticidal potential. These metabolites belong to diverse chemical classes such as phenalenones, xanthones, and pyrones, which have been implicated in deterrent or toxic effects when interacting with herbivorous insects in laboratory studies.

In plant–aphid systems, phytohormonal signaling not only regulates production of deterrent metabolites but also directs emission of volatile organic compounds (VOCs) that can influence plant–insect interactions. Herbivore-induced VOCs function as indirect signals that attract natural enemies of the herbivores and can be integral to Tri-trophic defense mechanisms in plants.

Interactions between endophytic fungi like *A. niger* and *A. vera* under aphid herbivory involve integration of microbial signals with plant hormonal pathways. Endophytic secondary metabolites produced by *A. niger* can contribute to the pool of bioactive molecules in plant tissues that are influenced by phytohormonal regulation and have implications for herbivore performance, although specific profiles for *A. vera* require direct characterization.

Research into plant use of endophytic fungi demonstrates that endophyte, mediated secondary metabolite production can alter plant nutritional and chemical landscapes in ways that reduce herbivory or pathogen colonization, contributing to plant resilience against insect attackers. In summary, recent advances in microbial signaling emphasize the roles of JA and SA signaling pathways (Table 5) in plant defensive responses to aphid herbivory, the rich secondary metabolite potential of endophytic fungi such as *Aspergillus nigera. Niger*, and the biochemical integration of plant and fungal signals that contribute to plant defense systems in *A. vera* ecosystems.

**Table 5:** Biological control and signaling pathways in pest management.

Biocontrol strategy	Mechanism	Advantages	Challenges	Reference
Enhancing microbial diversity	Promotes plant health, resistance, and soil health	Improves nutrient cycling, enhances plant resilience	Requires balance to avoid pathogen competition, needs management	Elnahal et al. (2022)
Inducing plant defenses	Use of JA (JA), VOCs	Strengthens natural plant defenses, reduces herbivory	Potential trade, offs with plant growth, energy costs for defense	Wei et al. (2020)
Signaling pathways (JA)	Activation of defense mechanisms, volatile compound release	Enhances pest resistance, induces natural predator attraction	Can be costly to the plant in terms of energy and growth	Noman et al. (2020) and Rapparini et al. (2024)
Signaling	Disrupts insect calcium channels, induces apoptosis	Enhances fungal virulence, accelerates pest death	Strain, specific, can affect non target organisms	Noman et al. (2020)
Quorum sensing signals	Modulates gut microbiome of herbivores, influences plant pest dynamics	Enhances interaction between plant and biocontrol agents	Complexity of microbial community dynamics can hinder efficacy	Rapparini et al. (2024)
Use of plant resistance genes	Gene editing to enhance plant resistance	Long term pest resistance, less reliance on chemical controls	Potential unintended effects on plant growth and ecosystem balance	Kessler et al. (2011)
Predator introduction	Release of natural predators (e.g., ladybugs, parasitoid wasps)	Natural pest suppression, environmentally friendly	Predator population control, may not be effective against all pests	Kessing et al. (2021)
Use of insecticidal proteins (Bt)	Use of genetically modified crops expressing Bt toxins	Effective against specific pests, low environmental impact	Resistance development in pests, off target effects on non pests	Tabashnik et al. (2020)
Use of plant volatiles	Release of volatile organic compounds (VOCs)	Attracts natural predators, repels pests	Can be resource intensive, species specific responses	Baldwin et al. (2022)
Biopesticide applications	Application of natural pathogens (e.g., bacteria, fungi)	Environmentally friendly, reduces pesticide use	Sensitive to environmental conditions, potential non, target effects	Stewart et al. (2020)
Microbial inoculation	Introducing beneficial microorganisms into soil or plants	Enhances plant health, suppresses pathogens	Risk of imbalance in microbial communities, inconsistent effects	Gray et al. (2019)
Nutrient, based control	Use of nutrient imbalances (e.g., high nitrogen levels) to disrupt pest growth	Low, cost, eco, friendly solution	Risk of promoting unwanted pests, nutrient leaching	Hartmann et al. (2023)
Chemical signaling manipulation	Disruption of pest communication (e.g., pheromones)	Disorients pests, reduces mating, and feeding	Requires high precision, may be costly to implement	Turchin et al. (2022)

## 6 Effects of abiotic factors on insect, fungus, plant interactions

Abiotic factors such as temperature, humidity, and soil conditions significantly influence the dynamics of interactions among *A. vera* (*A. vera*), herbivorous aphids, and the endophytic fungus *Aspergillus niger* by affecting plant physiology, endophytic colonization, secondary metabolism, and herbivore performance.

### 6.1 Temperature

Temperature strongly affects plant metabolic rates and the expression of defense pathways in response to herbivore attack. Elevated temperatures influence the biosynthesis and signaling of phytohormones such as jasmonic acid (JA) and salicylic acid (SA), which are central to *A. vera*’s defensive response to aphid herbivory. Phloem-feeding by aphids induces SA signaling and can modulate JA, dependent defenses, with both pathways responding to temperature variation in model systems (Gebretsadik et al., 2025). JA and SA signaling are known to regulate production of volatile organic compounds (VOCs) and secondary metabolites with defensive roles against herbivores (Gebretsadik et al., 2025; FEMS Microbiology Ecology). Temperature also alters the composition and activity of plant secondary metabolites, which are central to defense against aphid herbivory (Plant–aphid interactions review).

Temperature affects *A. niger* physiology and secondary metabolite production in culture systems, with metabolic profiles—such as organic acids and phenolic compounds—varying across temperature gradients in other fungi, which may also apply to *A. niger* because of its broad metabolic capacity (Frontiers in Microbiology). Endophytic fungi respond to abiotic temperature stress by adjustments in metabolite production that can influence plant stress physiology (MDPI microbiology reviews).

### 6.2 Humidity

Relative humidity influences plant water balance, stomatal conductance, and abiotic stress signaling. High humidity can reduce *A. vera* transpiration and maintain tissue turgor, affecting plant secondary metabolism and potential herbivore susceptibility (Fungal Endophytes as Mitigators Against Biotic and Abiotic Stresses). Humidity also influences endophytic fungal colonization; in many plant-endophyte systems, increased humidity affects fungal establishment and metabolite profiles, which, in turn, alter host plant chemistry (Plant use of endophytic fungi in defense).

Aphid populations are known to vary with humidity, with changes in insect feeding rates and population growth documented under different humidity regimes in other crop systems. Aphid performance and survival are influenced by humidity through effects on water balance and plant quality under stress (Plant–aphid interactions review).

### 6.3 Soil conditions

Soil properties, including nutrient availability, pH, and moisture—affect *A. vera* growth, the abundance of endophytic *A. niger*, and plant chemical composition associated with defense pathways. Fungal endophytes, including Aspergillus species, contribute to plant nutrient mineralization and phytohormone modulation under abiotic stress, which impacts host resilience (Fungal Endophytes as Mitigators against Biotic and Abiotic Stresses).

Endophytic colonization by fungi has been shown to modulate antioxidant enzyme activities and plant stress responses, which intersect with plant defense signaling against herbivory (Endophytic Fungi for Crops Adaptation to Abiotic Stresses). Endophytes can produce bioactive metabolites that directly or indirectly influence plant resistance to pests and may also affect soil microhabitat through altered rhizosphere interactions (Uniting the Role of Endophytic Fungi).

Soil moisture influences plant root function and foliar chemistry, with drought or high moisture conditions altering concentrations of defensive metabolites relevant to aphid resistance (Endophytic Fungi for Crops Adaptation to Abiotic Stresses).

### 6.4 Integration of abiotic effects on tri-trophic dynamics

Together, temperature, humidity, and soil quality influence *A. vera* biochemical pathways (e.g., JA and SA signaling), which regulate the production of defensive metabolites implicated in deterring aphid herbivory and mediating endophytic fungal interactions. Abiotic stresses alter endophytic fungal metabolism and plant secondary chemistry. These documented abiotic effects shape host plant physiology, endophytic fungal function, and herbivore performance, underscoring that environmental context is essential for understanding and managing plant–endomicrobe–aphid interactions in *A. vera* cultivation systems.

## 7 Agricultural application and pest management practices

Integrating biological control agents and endophytic fungi into *A. vera* cultivation provides a sustainable alternative to synthetic pesticides for managing aphid herbivores. Aphids such as *A. gossypii* are primary sap-feeding pests in agroecosystems and negatively affect crop productivity by reducing photosynthetic capacity, transmitting plant viruses, and triggering plant stress responses regulated by phytohormones including salicylic acid (SA) and jasmonic acid (JA) pathways (phytohormone defense roles).

Endophytic fungi—those that asymptomatically colonize plant tissues—are recognized for producing bioactive secondary metabolites that contribute to plant defense and can influence herbivore behavior or mortality. For example, *A. Niger* has been isolated from *A. vera* leaves at significant frequencies (≈13%) among endophytic communities, demonstrating its consistent association with *A. vera* tissues and suggesting a role in plant–microbe interactions. Endophytic fungal extracts, including those from *A. niger*, have been shown to exhibit larvicidal and insecticidal activities against agricultural pests in other plant systems, indicating the potential of fungal metabolites as alternative biocontrol agents.

The use of endophyte-derived compounds aligns with Integrated Pest Management (IPM) strategies, emphasizing reduced chemical input, ecological safety, and targeted pest suppression (endophytic fungi for crop protection). Fungal endophytes can produce a range of secondary metabolites—such as polyketides, alkaloids, and volatile organic compounds—that may deter herbivores directly or enhance host plant defense pathways, although specific characterization in *A. vera* remains limited to isolates and metabolite profiling research.

Colonization by endophytic fungi intersects with plant defense signaling. In general plant systems, herbivore attack triggers SA-regulated responses to phloem feeders and JA-associated signaling to insect damage, which regulate downstream defense gene expression and production of deterrent metabolites. The presence of fungal endophytes can modulate these host signaling pathways, influencing both constitutive and induced metabolite profiles that affect herbivore performance and overall plant health (endophytes modulate host defense).

Agricultural practices that support beneficial endophyte communities in the rhizosphere and phyllosphere-such as reduced tillage, organic amendments, and microbial inoculation—can enhance plant resilience to pests and environmental stressors. Diverse microbial communities improve nutrient cycling and can suppress soil-borne pathogens by competitive exclusion or by stimulating plant immune responses via induced systemic resistance (ISR) mechanisms that intersect with JA and SA signaling networks (endophyte benefits). Collectively, incorporating fungal endophytes like *A. niger* into *A. vera* production offers a multi, faceted biocontrol strategy: (i) direct insecticidal potential via metabolites with demonstrated bioactivity in other systems, (ii) modulation of host defense pathways involved in phytohormone signaling, and (iii) integration into broader IPM frameworks that reduce reliance on synthetic pesticides. To fully realize this potential in *A. vera*, further empirical research is needed to link specific metabolites and endophyte colonization with performance against aphid herbivores under field conditions.

## 8 Conclusion and future prospects

Fungal endophytes and plant signaling networks are central to sustainable pest management in agricultural systems. While the role of *Aspergillus nigera. Niger* as an endophyte in *A. vera* has been documented, and extracts from related fungal endophytes have demonstrated insecticidal activity in laboratory studies, the specific mechanisms by which *A. niger* influences aphid herbivores in *A. vera* require further elucidation through controlled studies.

Plant defense responses against aphids involve complex interactions between salicylic acid and jasmonic acid signaling pathways. These phytohormones regulate the expression of defense genes and the synthesis of secondary metabolites that deter or impair herbivory across many plant species. Understanding how endophytic colonization by fungi like *A. niger* intersects with these defense networks will be critical for developing biological control strategies tailored to *A. vera*.

Advances in multi-omics technologies—including genomics, transcriptomics, metabolomics, and metagenomics—can provide deeper insights into the biochemical and molecular basis of plant–endophyte–herbivore interactions. Next, generation sequencing (NGS) and metabolite profiling can reveal key gene clusters and metabolic pathways involved in endophyte colonization, secondary metabolite production, and modulation of plant defense responses, thereby aiding in the selection of effective biocontrol strains and formulations.

For agricultural applications, integrating biological approaches such as fungal endophytes into IPM systems will require field validated protocols that consider environmental variables, crop management practices, and compatibility with other beneficial organisms. As research continues to map the functional roles of endophyte taxa like *A. niger* in host plant defense and herbivore suppression, these insights will inform practical applications that improve crop resilience while reducing environmental impacts associated with conventional pesticides.
